# Enhancing intestinal anastomotic healing using butyrate: Systematic review and meta-analysis of experimental animal studies

**DOI:** 10.1371/journal.pone.0286716

**Published:** 2023-06-13

**Authors:** Aurelia C. L. Wildeboer, Claire P. M. van Helsdingen, Camille G. Gallé, Rob B. M. de Vries, Joep P. M. Derikx, Nicole D. Bouvy

**Affiliations:** 1 Department of Surgery, Maastricht University Medical Centre+ (MUMC+), Maastricht, The Netherlands; 2 Department of Pediatric Surgery, Emma Children’s Hospital, Amsterdam UMC, University of Amsterdam & Vrije Universiteit Amsterdam, Amsterdam, The Netherlands; 3 Tytgat Institute for Liver and Intestinal Research, Amsterdam Gastroenterology Endocrinology Metabolism, Amsterdam UMC, University of Amsterdam, Amsterdam, The Netherlands; 4 Department of General Surgery, Maastricht University, Maastricht, The Netherlands; 5 Systematic Review Centre for Laboratory Animal Experimentation (SYRCLE), Department for Health Evidence, Radboud Institute for Health Sciences, Radboud University Medical Center, Nijmegen, The Netherlands; Université de Montréal: Universite de Montreal, CANADA

## Abstract

**Background:**

Despite advancements in surgical technique and perioperative care, intestinal anastomoses still have a 10–15 per cent risk of leakage, which results in considerable morbidity and/or mortality. Recent animal studies have suggested that administration of butyrate to the anastomotic site results in enhanced anastomotic strength, which may prevent leakage. This systematic review and meta-analysis summarises current evidence concerning the effect of butyrate administration on anastomotic healing and will form a scientific basis for the development of new research into this subject.

**Methods:**

Animal studies on the effect of butyrate-based interventions in models of intestinal anastomotic healing were systematically retrieved from online databases. Bibliographical data, study characteristics and outcome data were extracted, and internal validity of the studies was assessed. Outcomes studied through meta-analysis concerned: anastomotic strength, anastomotic leakage, collagen metabolism and general histologic parameters of wound healing.

**Results:**

A comprehensive search and selection identified 19 relevant studies containing 41 individual comparisons. Design and conduct of most experiments were poorly reported resulting in an unclear risk of bias. Meta-analyses showed that butyrate administration significantly increases anastomotic strength (SMD 1.24, 0.88 to 1.61), collagen synthesis (SMD 1.44, 0.72 to 2.15) and collagen maturation, making anastomoses less prone to leakage in the early postoperative period (OR 0.37, 0.15 to 0.93).

**Conclusion:**

This systematic review and meta-analysis shows that there is potential ground to investigate the use of butyrate in clinical trials to prevent anastomotic leakage in intestinal surgery. However, more research is necessary to define the best application form, dosage and administration route.

## Introduction

Intestinal anastomoses are one of the most frequently performed procedures in abdominal surgery [1, 2]. Anastomoses are often constructed after gastrointestinal resections, which are performed for malignant or benign causes (ischemia, perforation or obstruction) [2]. Healing of this newly constructed surgical connection is a complex pathophysiological process, and disturbance of anastomotic healing may lead to surgical complications, such as anastomotic leakage (AL) [3]. In AL, spillage of enteric content into the sterile abdominal cavity frequently results in severe morbidity and/or even mortality [4]. Colorectal carcinoma patients, in addition, have an increased risk of local recurrence and decreased disease-free survival after AL, which negatively impacts their oncologic prognosis [5]. Despite advancements in surgical technique and perioperative care, intestinal anastomoses still have an average 10–15 per cent risk of leakage [4, 6–8].

In order to reduce AL incidence, numerous (pre-)clinical studies have attempted to improve anastomotic healing, but have failed to demonstrate efficacy in clinical practice. Neither the identification of risk factors in order to facilitate proper patient selection, nor the development of anastomotic devices to reinforce the anastomotic site have shown promise so far. With the incidence of AL remaining the same as it was 50 years ago [9], the need to find a solution for anastomotic failure remains. Previous animal studies, however, have suggested that administration of butyrate to the anastomotic site results in enhanced anastomotic strength [7, 9, 10], which may prevent leakage. Butyrate, a bacterial fermentation product, is one of the predominant short chain fatty acids (SCFAs) and mainly serves as colonocyte fuel [10]. This bacterial metabolite also demonstrated remarkable regenerative and anti-inflammatory effects improving damaged intestinal mucosa in an animal model of colitis [11, 12]. Butyrate’s regenerative potential is thought to stem from its ability to stimulate reepithelialisation and influence collagen lysis by reducing matrix metalloproteinase (MMP) release [13]. Knowing that (1) the relative abundance of butyrate producing bacteria and concomitant endoluminal butyrate concentrations can be low in CRC patients [14, 15], (2) luminal nutrients like butyrate contribute to more than 70 per cent of the energy supply for the intestine [16], and (3) anastomotic healing is metabolically quite demanding [17]; it can be hypothesized that the administration of butyrate can be of benefit to anastomotic healing.

Nevertheless, there are currently no butyrate-based interventions that have found their way into clinical trials on AL [18], which delays translation into patient care. A first step in this process should therefore be to rigorously assess all existing relevant animal evidence on this topic and justify the grounds for translational research before beginning any new research. Hence, we have conducted a systematic review regarding the effects of butyrate-based interventions on anastomotic healing in animals to obtain insight in into the potential clinical usefulness of butyrate for the prevention of anastomotic failure. This systematic review will subsequently form a scientific basis, and guide, for the development of new (clinical) research into the subject.

## Methods

This systematic review and meta-analysis of animal studies was reported in accordance with the PRISMA guidelines [19]. A protocol was registered at the International Prospective Register of Systematic Reviews (PROSPERO)(registration number CRD42020210683).

### Eligibility criteria

Articles in all languages concerning in vivo experimental animal studies on the effect of butyrate-containing applications on the healing of intestinal anastomoses, or transmural defects, were considered eligible. Conference abstracts and review articles, all human studies, in vitro or ex vivo studies, all studies on anastomoses other than intestinal (for example vascular or urological), studies only reporting outcomes not related to anastomotic healing (such as cancer recurrence), studies without a control group, and those in which no butyrate-containing applications were used were excluded. No discrimination was made between normal/healthy anastomotic healing models and impaired healing models. A detailed list of the selection criteria is provided in the online registered PROSPERO protocol.

### Search strategy

A systematic search was conducted in PubMed and the OvidSP version of Embase using a structured search query to cover any study on intestinal anastomoses and the use of butyrate (S1 Table). Additional to the bibliographical databases, ResearchGate was also searched. Search terms were identified by reviewing Medical Subject Headings (MeSH) for appropriate search terms and extracting keywords from primary data studies belonging to a test set of potentially relevant articles that was created during the composition of the review protocol. To ensure search terms retrieved all studies in the test set, a test search was conducted before the definitive search. The date of last systematic search was 8 August 2022. Any publication to this date meeting the eligibility criteria was considered for inclusion. Cross-references from the included articles were manually searched for additional papers. No limits were placed on year of publication or language. All search results were downloaded and electronically managed using EndNote X9^TM^ (Clarivate Analytics, Philadelphia, Pennsylvania, USA).

### Study selection

After removing duplicates, all references were imported into Rayyan [20] (http://rayyan.qcri.org) for title and abstract screening. References were screened by two authors (AW and CvH) independently. Any disagreement was resolved by discussion and consensus. Retrieval of relevant full-text articles was attempted through online searching, consulting Dutch university libraries, or by requesting a corresponding author. All full-text articles were subjected to independent reviewing by two authors (AW and CvH) according to the eligibility criteria.

### Data extraction and management

Included studies were subjected to data extraction by one reviewer (AW) using a predefined data extraction template in Excel®. Extracted data included general characteristics of the experimental model (species, sex, total number of animals used, experimental groups, control groups), more specific information regarding the surgical procedure (intestinal segment involved, type of anastomotic construction, surgical technique), details on the investigated compound (timing of administration, dose, route of administration), additional interventions, and outcome measures.

To evaluate the effect of butyrate on anastomotic healing the following outcome domains were defined a priori [21, 22] and refined during data extraction: (1) indicators of mechanical anastomotic strength (e.g. anastomotic bursting pressure, bursting site); (2) macroscopic indicators of anastomotic failure or other complications (e.g. anastomotic leakage/dehiscence, adhesions); (3) biochemical and histological indicators of anastomotic healing (e.g. hydroxyproline content, inflammatory cytokine production, generic histological examination). From each of the independent comparisons the outcome data related to the three domains of anastomotic healing was identified. Subsequently group averages (mean, median or incidence), standard deviation (SD), standard error (SE) or ranges and number on animals per specific group (n) were collected or where necessary recalculated. Graphical outcome data was estimated using an on-screen ruler (WebPlotDigitizer 4.5, Automeris, Pacifica, CA, USA) [23]. Study results, as reported by the authors, were also extracted and categorized under the three aforementioned outcome domains for a comprehensive overview of the reported effects of butyrate administration on anastomotic healing. Study effects were indicated as having positive (for example ‘butyrate increases bursting pressure’), neutral (for example ‘butyrate does not affect the incidence of AL’) or negative (for example ‘butyrate decreases hydroxyproline content’) effect on anastomotic healing according to authors’ conclusions. All of the extracted data was checked for accuracy, consistency and completeness by two independent reviewers (CvH and CG).

### Data synthesis and analysis

Outcomes expressing the effect of butyrate on anastomotic healing that were reported in at least three independent studies were pooled for meta-analysis using Comprehensive Meta-Analysis (CMA version 3). First, we calculated the effect sizes for continuous outcomes (Hedges G) and dichotomous outcomes (Odds Ratio) for each of the individual comparisons. If studies reported data on multiple cohorts (e.g. different types of butyrate-containing applications used in one study or different anastomotic models), these were included in the meta-analysis as independent comparisons. When multiple experimental groups were compared to the same control group, group size of the control group was divided by the number of comparisons made. In case of a zero in the events or control group, ‘1’ was added to each cell to calculate the OR (Odds Ratio). Subsequently we conducted meta-analyses for at least one outcome per domain. Despite the anticipated heterogeneity, the individual effect sizes were pooled to obtain an overall Hedges G, or OR, and a 95% confidence interval (c.i.). We used the random effects model which takes both the precision of individual studies and the variation between studies into account and weights each study accordingly. Heterogeneity among studies was quantified using *I*^*2*^. Subgroups were predefined and registered in the online registered PROSPERO protocol. Subgroup analyses were planned for anastomotic model (AH or IAH) and timing of the outcome analysis (a; early phase of anastomotic wound healing or b; late phase of anastomotic healing). Other factors, like form of butyrate, dosage or administration route turned out to yield subgroup sizes that were too small to conduct meaningful analyses. Results of subgroup analyses were only interpreted when subgroups contained at least data from five independent studies or ten comparisons per subgroup. We expected the variance to be comparable within the subgroups; therefore, we assumed a common among-study variance across subgroups. For subgroup analyses, we adjusted our significance level according to the conservative Bonferroni method to account for multiple analyses (p* number of comparisons). Nevertheless, differences between subgroups should be interpreted with caution and should only be used for constructing new hypotheses rather than for drawing final conclusions. To determine the robustness of the analyses, sensitivity analysis was conducted. First the effect of the administration of different forms of butyrate was investigated. Secondly, we assessed the effect of in and excluding studies with zero counts in the denominator for the meta-analyses concerning incidences. Evidence for publication bias was deducted from Funnel plots and Trim and Fill analysis. Because SMDs may cause funnelplot distortion, the SMD was plotted against a sample size-based precision estimate.

### Study quality assessment

Risk of bias was assessed independently by two authors (AW and CvH) using SYRCLE’s risk of bias tool for animal studies [24]. The evaluated domains included the random and blinded sequence generation (selection bias), blinding of animal caregivers and researchers (performance bias), blinding of outcome assessment (detection bias), handling of incomplete outcome data (attrition bias), and selective reporting of outcomes (reporting bias). A ‘yes’ score indicates low risk of bias; a ‘no’ score indicates high risk of bias; and a ‘?’ score indicates an unclear risk of bias. To account for the usually poor reporting of experimental details in animal studies [25], three additional items were scored only on reporting (any level): reporting of any measure of randomisation, reporting of any measure of blinding, and reporting of a power calculation. For these three items, a ‘yes’ score indicates ‘reported’, and a ‘no’ score indicates ‘not reported’. Any disagreements during assessment was resolved by discussion between the two authors.

## Results

**Fig 1:**
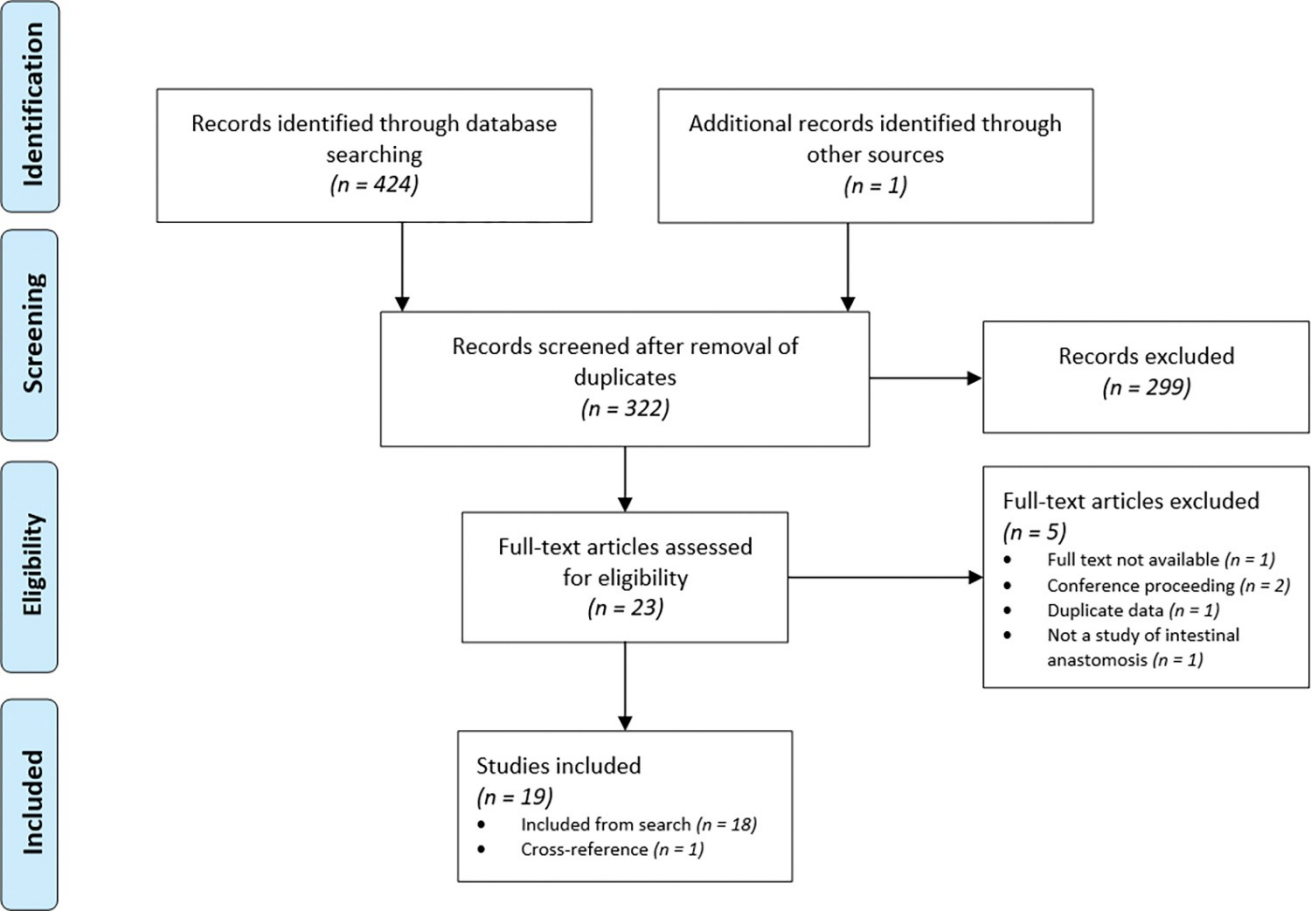
PRISMA flowchart of the study selection process.

Searching in databases resulted in 424 records and hand-searching of ResearchGate provided one additional article [26]. After removing duplicates, 322 records remained. Title and abstract screening yielded 23 studies, of which 18 [7, 9, 10, 16, 26–39] were included for this review after reading the full-text; one additional article [40] was later found through scrutinising cross-references, resulting in a total of 19 included studies containing 41 individual comparisons (Fig 1).

### Study characteristics

**Fig 2:**
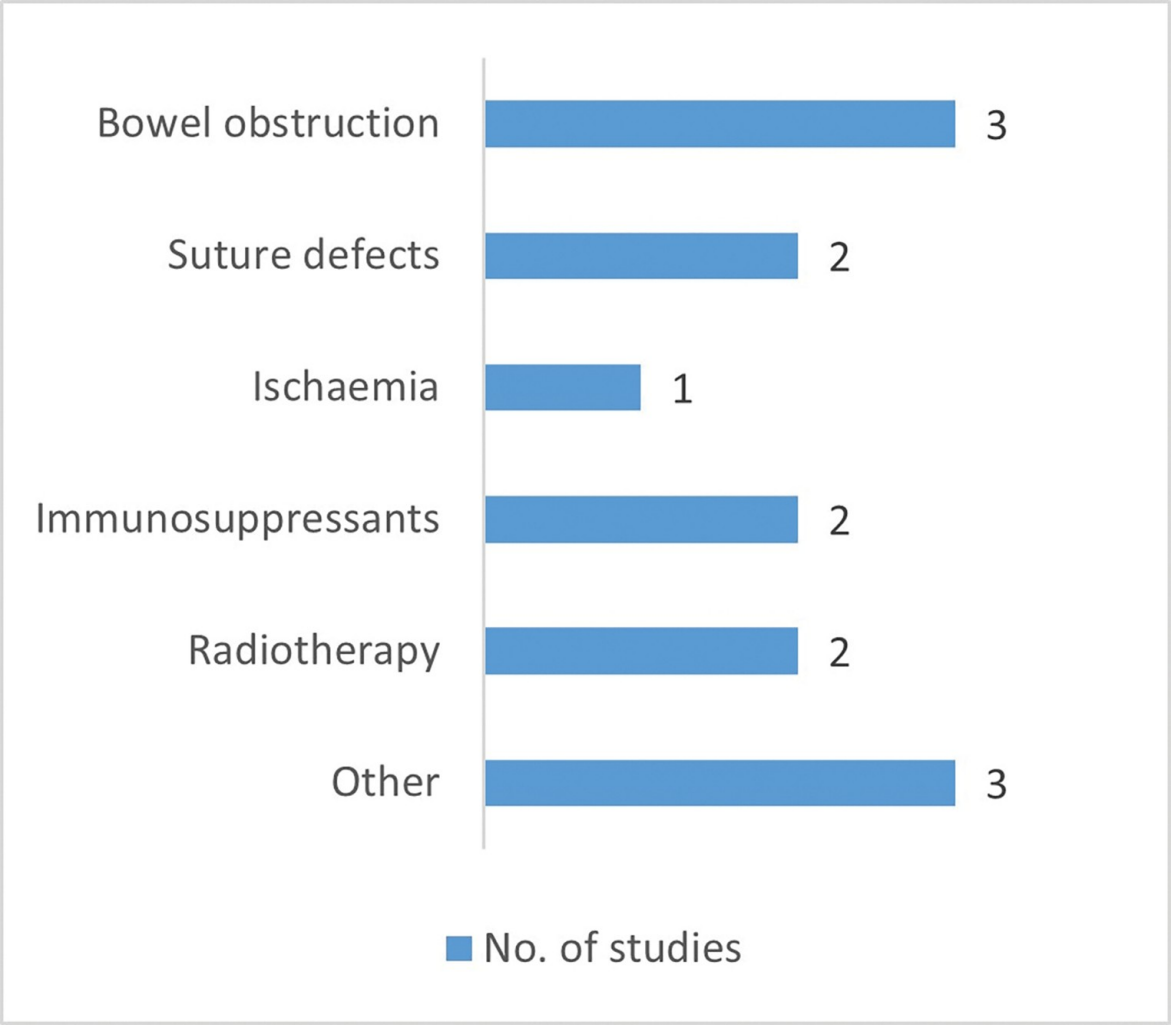
Number of studies included concerning impaired anastomotic healing, divided into the different categories of IAH. Categories of methods to impair anastomotic healing (with examples in parentheses): bowel obstruction (transient ligature [27, 28, 30]); suture defects (reduced amount of sutures [7, 9]); ischaemia (marginal artery clamping [39]); immunosuppressants (tacrolimus [29], corticosteroids [16]); radiotherapy (various regimens [32, 38]); other (reduction of endogenous butyrate production through cecectomy/cecostomy and/or fibre-free diet [34–36]).

The general characteristics of the included studies are summarised in Table 1. All included studies conducted their experiments in rat models (1 300 animals in total) of intestinal anastomosis. Six studies [2, 4, 9, 24, 34, 37] used normal intestinal anastomotic healing (AH) models, nine used models [7, 9, 27–30, 34–36] of impaired anastomotic healing (IAH) and four studies [16, 32, 38, 39] employed both models. The methods that were used to impair anastomotic healing are shown in Fig 2. Most anastomoses were located in the distal part of the large intestine and had a sutured end-to-end configuration. At least five different forms of butyrate were identified, of which the SCFA mixture (acetate, propionate and butyrate) was used the most (9 studies). The preferred delivery method for butyrate was colonic irrigation through either intracolonic lavage (usually intraoperative) or enema (both pre- and postoperative administrations). Dosing schedule and administration route differed to some extent between studies (between the different forms of butyrate but also within a butyrate group).

**Table 1:** Characteristics table.

Reference	Animal model					Intervention					
ID	Strain	Sex	n	Site	Model	Compound	Dosage	Frequency	Duration	Delivery	Timing
Aguilar, 1995 (IAH-a)	Wistar	M	108	Distal colon	IAH	Butyrate*	40 mM	Single dose	n.a.	Lavage	Intraoperative
Aguilar, 1995 (IAH-b)				Distal colon	IAH	Butyrate*	40 mM	Single dose	n.a.	Lavage	Intraoperative
Aguilar, 1997 (IAH-a)	Wistar	M	107	Distal colon	IAH	Butyrate*	60 mM	Single dose	n.a.	Lavage	Intraoperative
Aguilar, 1997 (IAH-b)				Distal colon	IAH	Butyrate*	60 mM	Single dose	n.a.	Lavage	Intraoperative
Akbulut, 2019 (IAH-b)	SD	M	48	Distal colon	IAH	AGHMB	685 mg/kg	Daily	21 days	SPO	Perioperative
Bloemen, 2010 (IAH-a)	Wistar	M	54	Distal colon	IAH	Butyrate	60 mM	Daily	3 days	Enema	Postoperative
Bloemen, 2010 (IAH-b)				Distal colon	IAH	Butyrate	60 mM	Daily	7 days	Enema	Postoperative
Bosmans, 2017 (1) (IAH-b)	Wistar	M	84	Proximal colon	IAH	Butyrin	20 massa%	Single dose	7 days	Patch	Perioperative
Bosmans, 2017 (2) (IAH-b)				Proximal colon	IAH	HA-butyrate	60 mM	Single dose	n.a.	LI	Intraoperative
Bosmans, 2017 (3a) (IAH-b)				Distal colon	IAH	HA-butyrate	60 mM	Daily	7 days	Enema	Postoperative
Bosmans, 2017 (3b) (IAH-b)				Distal colon	IAH	Butyrate	60 mM	Daily	7 days	Enema	Postoperative
Erbil, 2000 (IAH-a)	Wistar	M	144	Distal colon	IAH	Butyrate*	40 mM	Single dose	n.a.	Lavage	Intraoperative
Erbil, 2000 (IAH-b)				Distal colon	IAH	Butyrate*	40 mM	Single dose	n.a.	Lavage	Intraoperative
Fa-Si-Oen, 2006 (AH-b)	SD	F	70	Distal colon	AH	Butyrate	3 mmol	Single dose	n.a.	LI+PI	Intraoperative
Greca, 2003 (1) (AH-b)	Wistar	NA	50	Distal colon	AH	Butyrate	80 mM	Daily	7 days	Enema	Postoperative
Greca, 2003 (2) (AH-b)				Distal colon	AH	Butyrate	80 mM	Daily	14 days	Enema	Postoperative
Kerem, 2006 (AH-a)	Wistar	NA	84	Distal colon	AH	Butyrate*	20 mM	Daily	5 days	Enema	Preoperative
Kerem, 2006 (AH-b)				Distal colon	AH	Butyrate*	20 mM	Daily	5 days	Enema	Preoperative
Kerem, 2006 (IAH-a)				Distal colon	IAH	Butyrate*	20 mM	Daily	5 days	Enema	Preoperative
Kerem, 2006 (IAH-b)				Distal colon	IAH	Butyrate*	20 mM	Daily	5 days	Enema	Preoperative
Kusabbi, 2015 (post) (AH-a)	Wistar	M	70	Distal colon	AH	AGHMB	300 mg/kg**	Daily	3 days	SPO	Postoperative
Kusabbi, 2015 (peri) (AH-a)				Distal colon	AH	AGHMB	300 mg/kg**	Daily	10 days	SPO	Perioperative
Kusabbi, 2015 (post) (AH-b)				Distal colon	AH	AGHMB	300 mg/kg**	Daily	7 days	SPO	Postoperative
Kusabbi, 2015 (peri) (AH-b)				Distal colon	AH	AGHMB	300 mg/kg**	Daily	14 days	SPO	Perioperative
Matthew, 2010 (IAH-b)	Wistar	M	40	Distal colon	IAH	Butyrate	80 mM	Daily	4 days	Enema	Postoperative
Netta, 2010 (3) (AH-a)	Wistar	M	40	Distal colon	AH	Butyrate*	NA	Daily	11 days	SPO	Perioperative
Netta, 2010 (4) (AH-a)				Distal colon	AH	Butyrate*	60 mM	Single dose	n.a.	Lavage	Intraoperative
Netta, 2014 (AH-a)	Wistar	M	50	Distal colon	AH	Butyrate*	60 mM	Single dose	n.a.	Lavage	Intraoperative
Netta, 2014 (IAH-a)				Distal colon	IAH	Butyrate*	60 mM	Single dose	n.a.	Lavage	Intraoperative
Rolandelli, 1986 (IAH-b)	SD	M	33	Distal colon	IAH	Butyrate*	20 mM	Continuous	6 days	LI	Postoperative
Rolandelli, 1997 (IAH-b)	SD	M	28	Distal colon	IAH	Butyrate	130 mM	Daily	5 days	IV	Postoperative
Seker, 2013 (AH-b)	Wistar	M	50	Proximal colon	AH	AGHMB	208 mg/kg**	Daily	7 days	SPO	Postoperative
Terzi, 2004 (IAH-a)	Wistar	M	60	Distal colon	IAH	Butyrate*	40 mM	Daily	5 days	Enema	Preoperative
Terzi, 2004 (IAH-b)				Distal colon	IAH	Butyrate*	40 mM	Daily	5 days	Enema	Preoperative
Terzi, 2004 (AH-a)				Distal colon	AH	Butyrate*	40 mM	Daily	5 days	Enema	Preoperative
Terzi, 2004 (AH-b)				Distal colon	AH	Butyrate*	40 mM	Daily	5 days	Enema	Preoperative
Topcu, 2002 (IAH-a)	Wistar	NA	160	Distal colon	IAH	Butyrate*	20 mM	Single dose	n.a.	Enema	Intraoperative
Topcu, 2002 (IAH-b)				Distal colon	IAH	Butyrate*	20 mM	Single dose	n.a.	Enema	Intraoperative
Topcu, 2002 (AH-a)				Distal colon	AH	Butyrate*	20 mM	Single dose	n.a.	Enema	Intraoperative
Topcu, 2002 (AH-b)				Distal colon	AH	Butyrate*	20 mM	Single dose	n.a.	Enema	Intraoperative
Yaman, 2012 (AH-b)	Wistar	M	20	Distal colon	AH	AGHMB	685 mg/kg	Daily	7 days	SPO	Preoperative

* Model: AH = normal anastomotic healing, IAH = impaired anastomotic healing. *n* = Total number of animals used in the study. NA = information not available. N.a. = not applicable. Butyrate* = mixture of SCFAs containing acetate, propionate and butyrate. AGHMB = mixture of arginine, glutamine and hydroxymethylbutyrate. Delivery categories (with examples in parentheses): lavage (intracolonic irrigation); SPO = supplemented per os (dietary supplement or via gavage); enema (transrectal irrigation through gauge or retention catheter); patch (drug-eluting patch); LI = local infusion (through syringe directly in intestinal lumen, peritoneal cavity or ostomy); PI = peritoneal injection; IV = intravenous administration (total parenteral nutrition).

The vast majority of studies reported on the effect of butyrate supplementation on anastomotic healing using outcomes from all three previously defined domains (Table 2). Most commonly reported outcomes concerning mechanical strength of the anastomosis were: anastomotic bursting pressure (BPR) and bursting site (BS). Other less frequent reported outcomes in this domain were anastomotic circumference (AC) and bowel wall tension or bursting wall tension (BWT). In the second outcome domain the most commonly reported outcomes were: anastomotic leakage (AL) and adhesions. Some studies also described more general complications (e.g. laparotomy wound infection, intestinal obstruction and weight loss). The last domain, concerning biochemical and histological indicators of anastomotic healing, was represented by hydroxyproline (HP) content and general histologic evaluation parameters of wound healing (e.g. reepithelialisation, inflammatory cell infiltration, fibroblast proliferation, neovascularization). Other reported outcomes involved derivatives of collagen metabolism other than HP (e.g. collagen ratios), matrix metalloproteinase (MMP) activity and cytokine levels (e.g. TNF-α, TGF-β). Timing of outcome assessments ranged from 3 to 14 days after surgery for all outcomes. Eleven studies assessed their outcomes on only one time point, of which two [10, 16] within the first 4 days after surgery (a), and nine [4, 5, 13, 19, 22, 26, 29, 32, 37] on day 5 or later (b). Eight studies [7, 27, 28, 30, 32, 33, 38, 39] performed outcome assessments in both time frames (a, b).

**Table 2:** Anastomotic healing outcomes.

Reference	Outcomes			
ID	Timing of outcome assessment	1	2	3
**Aguilar, 1995 (IAH-a)**	a (3)	BPR ↔, BWT ↔, Bursting in suture line ↔	Anastomotic leakage ↔	Hydroxyproline ↔
**Aguilar, 1995 (IAH-b)**	b (6)	BPR ↔, BWT ↔, Bursting in suture line ↓	Anastomotic leakage ↔	Hydroxyproline ↑
**Aguilar, 1997 (IAH-a)**	a (3)			Reepithelialisation ↔, Crypt depth ↑
**Aguilar, 1997 (IAH-b)**	b (6)			Reepithelialisation ↑, Crypt depth ↔
**Akbulut, 2019 (IAH-b)**	b (7)	BPR ↑	Anastomotic leakage ↔, Adhesions ↔	Inflammatory cell infiltration ↔, Collagen deposition ↔, Neovascularisation ↑, Fibroblast proliferation ↑, TGF-β1 ↔
**Bloemen, 2010 (IAH-a)**	a (3)	BPR ↔	Anastomotic leakage ↔, Adhesions ↔	Collagen deposition ↔, Mature collagen/immature collagen ↑, Inflammatory cell infiltration ↔, Fibroblast proliferation ↔, Reepithelialisation ↔, MMP ↑
**Bloemen, 2010 (IAH-b)**	b (7)	BPR ↑	Anastomotic leakage ↔, Adhesions ↔	Collagen deposition ↔, Mature collagen/immature collagen ↔, Inflammatory cell infiltration ↔, Fibroblast proliferation ↔, Reepithelialisation ↔, MMP ↑
**Bosmans, 2017 (1) (IAH-b)**	b (7)		Anastomotic leakage ↔, Adhesions ↔	MMP ↔, Mucin ↔, Collagen deposition ↔, Mature collagen/immature collagen ↔, Inflammatory cell infiltration ↔, Fibroblast proliferation ↔
**Bosmans, 2017 (2) (IAH-b)**	b (7)	BPR ↔	Anastomotic leakage ↔, Adhesions ↔	MMP ↔, Mucin ↔, Collagen deposition ↔, Mature collagen/immature collagen ↔, Inflammatory cell infiltration ↔, Fibroblast proliferation ↔
**Bosmans, 2017 (3a) (IAH-b)**	b (7)	BPR ↑	Anastomotic leakage ↓, Adhesions ↔	MMP ↔, Mucin ↔, Collagen deposition ↔, Mature collagen/immature collagen ↔, Inflammatory cell infiltration ↔, Fibroblast proliferation ↔
**Bosmans, 2017 (3b) (IAH-b)**	b (7)	BPR ↑	Anastomotic leakage ↓, Adhesions ↔	MMP ↔, Mucin ↔, Collagen deposition ↔, Mature collagen/immature collagen ↔, Inflammatory cell infiltration ↔, Fibroblast proliferation ↔
**Erbil, 2000 (IAH-a)**	a (3)	BPR ↑	Anastomotic leakage ↔	Hydroxyproline ↑
**Erbil, 2000 (IAH-b)**	b (6)	BPR ↑	Anastomotic leakage ↔	Hydroxyproline ↑
**Fa-Si-Oen, 2006 (AH-b)**	b (5)	BPR ↔	Anastomotic leakage ↔	
**Greca, 2003 (1) (AH-b)**	b (7)		Anastomotic leakage ↔, Adhesions ↔	Collagen maturation ↑, Total collagen ↔
**Greca, 2003 (2) (AH-b)**	b (14)		Anastomotic leakage ↔, Adhesions ↔	Collagen maturation ↑, Total collagen ↔
**Kerem, 2006 (AH-a)**	a (3)	BPR ↔	Anastomotic leakage ↔, Adhesions ↔	Hydroxyproline ↔, MMP ↔, Reepithelialisation ↔, Muscularis propria regeneration ↔, Necrosis ↔, Exudate ↔, Inflammatory cell infiltration ↔, Fibroblasts ↔, Granulation tissue ↔
**Kerem, 2006 (AH-b)**	b (7)	BPR ↔	Anastomotic leakage ↔, Adhesions ↔	Hydroxyproline ↔, MMP ↔, Reepithelialisation ↔, Muscularis propria regeneration ↔, Necrosis ↔, Exudate ↔, Inflammatory cell infiltration ↔, Fibroblasts ↔, Granulation tissue ↔
**Kerem, 2006 (IAH-a)**	a (3)	BPR ↑	Anastomotic leakage ↔, Adhesions ↔	Hydroxyproline ↑, MMP ↓, Reepithelialisation ↔, Muscularis propria regeneration ↔, Necrosis ↓, Exudate ↔, Inflammatory cell infiltration ↔, Fibroblasts ↔, Granulation tissue ↑
**Kerem, 2006 (IAH-b)**	b (7)	BPR ↑	Anastomotic leakage ↔, Adhesions ↔	Hydroxyproline ↑, MMP ↓, Reepithelialisation ↑, Muscularis propria regeneration ↔, Necrosis ↓, Exudate ↓, Inflammatory cell infiltration ↑, Fibroblasts ↑, Granulation tissue ↑
**Kusabbi, 2015 (post) (AH-a)**	a (3)	BPR ↑	Anastomotic leakage ↔, Adhesions ↔	Hydroxyproline ↑, Collagen deposition ↑, Inflammatory cell infiltration ↑, Fibroblasts ↑, neovascularisation ↑
**Kusabbi, 2015 (pre+post) (AH-a)**	a (3)	BPR ↑	Anastomotic leakage ↔, Adhesions ↔	Hydroxyproline ↑, Collagen deposition ↑, Inflammatory cell infiltration ↑, Fibroblasts ↑, neovascularisation ↑
**Kusabbi, 2015 (post) (AH-b)**	b (7)	BPR ↑	Anastomotic leakage ↔, Adhesions ↔	Hydroxyproline ↑, Collagen deposition ↑, Inflammatory cell infiltration ↑, Fibroblasts ↔, neovascularisation ↑
**Kusabbi, 2015 (pre+post) (AH-b)**	b (7)	BPR ↑	Anastomotic leakage ↔, Adhesions ↔	Hydroxyproline ↑, Collagen deposition ↑, Inflammatory cell infiltration ↑, Fibroblasts ↔, neovascularisation ↔
**Matthew, 2010 (IAH-b)**	b (7)	BPR ↔, BWT ↑, AC ↔	Anastomotic leakage ↔	
**Netta, 2010 (3) (AH-a)**	a (4)	BPR ↔, Bursting in suture line ↔	Anastomotic leakage ↔, Adhesions ↔	TNF-α ↔
**Netta, 2010 (4) (AH-a)**	a (4)	BPR ↑, Bursting in suture line ↔	Anastomotic leakage ↓, Adhesions ↓	TNF-α ↓
**Netta, 2014 (AH-a)**	a (4)	BPR ↑		TNF-α ↔
**Netta, 2014 (IAH-a)**	a (4)	BPR ↔		TNF-α ↓
**Rolandelli, 1986 (IAH-b)**	b (6)	BPR ↑, BWT ↑, AC ↔, Bursting in suture line ↓	Anastomotic leakage ↔	Hydroxyproline ↔
**Rolandelli, 1997 (IAH-b)**	b (5)	BPR ↑, BWT ↑, AC ↔	Anastomotic leakage ↔	Hydroxyproline ↔
**Seker, 2013 (AH-b)**	b (7)	BPR ↑		Hydroxyproline ↑
**Terzi, 2004 (IAH-a)**	a (3)	BPR ↑, Bursting in suture line ↔	Anastomotic leakage ↔, Adhesions ↔	Collagen deposition ↔, Apposition of mucosal and muscular wound edges ↔, Reepithelialisation ↔, Muscularis propria regeneration ↔, Necrosis ↓, Exudate ↔, Inflammatory cell infiltration ↔, Fibroblasts ↔, Granulation tissue ↔
**Terzi, 2004 (IAH-b)**	b (7)	BPR ↑, Bursting in suture line ↔	Anastomotic leakage ↔, Adhesions ↔	Collagen deposition ↔, Apposition of mucosal and muscular wound edges ↔, Reepithelialisation ↔, Muscularis propria regeneration ↔, Necrosis ↓, Exudate ↓, Inflammatory cell infiltration ↔, Fibroblasts ↔, Granulation tissue ↑
**Terzi, 2004 (AH-a)**	a (3)	BPR ↔, Bursting in suture line ↔ (outside)	Anastomotic leakage ↔, Adhesions ↔	Collagen deposition ↔, Apposition of mucosal and muscular wound edges ↔, Reepithelialisation ↔, Muscularis propria regeneration ↔, Necrosis ↔, Exudate ↔, Inflammatory cell infiltration ↔, Fibroblasts ↔, Granulation tissue ↔
**Terzi, 2004 (AH-b)**	b (7)	BPR ↔, Bursting in suture line ↔ (outside)	Anastomotic leakage ↔, Adhesions ↔	Collagen deposition ↔, Apposition of mucosal and muscular wound edges ↔, Reepithelialisation ↑, Muscularis propria regeneration ↔, Necrosis ↔, Exudate ↔, Inflammatory cell infiltration ↑, Fibroblasts ↔, Granulation tissue ↑
**Topcu, 2002 (IAH-a)**	a (4)	BPR ↑, Bursting in suture line ↔	Anastomotic leakage ↓, Adhesions ↓	
**Topcu, 2002 (IAH-b)**	b (7)	BPR ↑, Bursting in suture line ↓	Anastomotic leakage ↓, Adhesions ↓	
**Topcu, 2002 (AH-a)**	a (4)	BPR ↔, Bursting in suture line ↔	Anastomotic leakage ↔, Adhesions ↔	
**Topcu, 2002 (AH-b)**	b (7)	BPR ↔, Bursting in suture line ↓	Anastomotic leakage ↔, Adhesions ↔	
**Yaman, 2012 (AH-b)**	b (7)	BPR ↑	Anastomotic leakage ↔	Hydroxyproline ↑, Collagen deposition ↑, Reepithelialisation ↑, Inflammatory cell infiltration ↔, Fibroblast proliferation ↔, neovascularisation ↔

٭Reported outcome domain: 1, indicators of mechanical anastomotic strength; 2, macroscopic indicators of anastomotic failure or other complications; 3, biochemical and histological indicators of anastomotic healing. Timing of outcome assessment (with the exact day in parentheses): a = sooner than five days after surgery, b = five days after surgery or later. BPR = bursting pressure. BWT = bursting wall tension or bowel wall tension. AC = anastomotic circumference.

### Reporting quality and risk of bias

**Fig 3:**
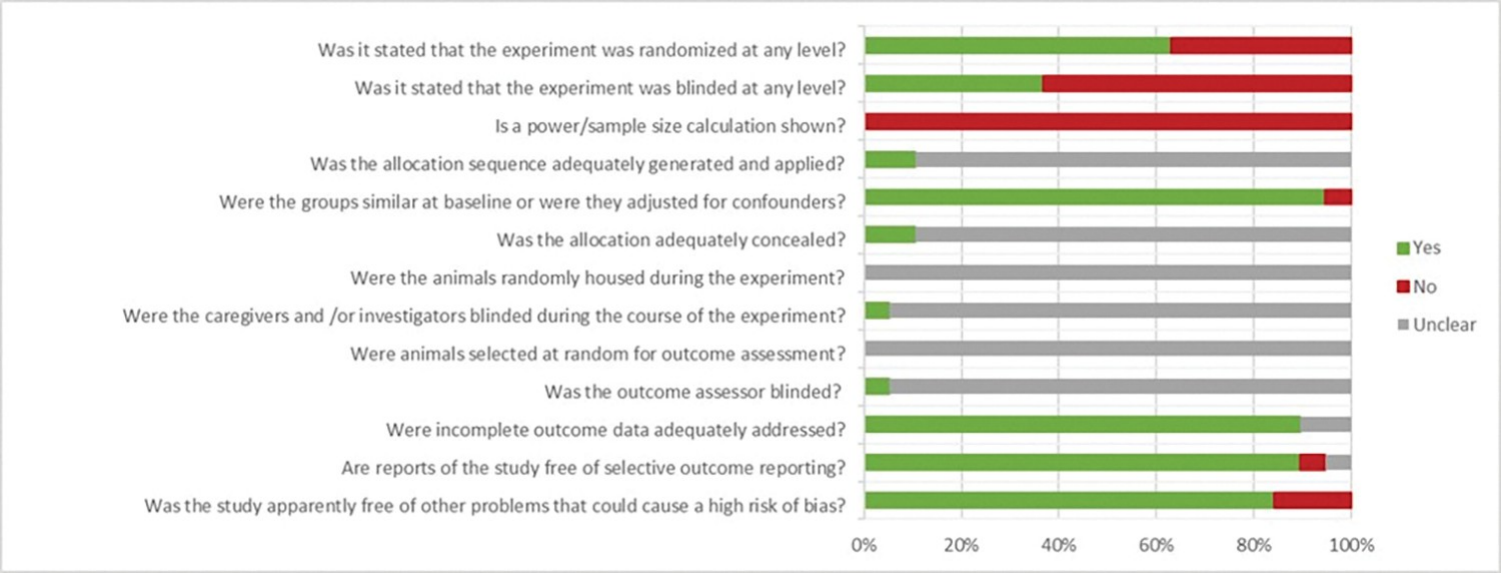
Results of the risk of bias assessment of the 19 studies included in this systematic review. The first three items assess study quality by scoring reporting, a ‘yes’ score indicating reported, and a ‘no’ score indicating not reported. The other items assessed risk of bias, with ‘yes’ indicating low risk of bias, ‘no’ high risk of bias and ‘?’ unclear risk of bias.

Results of the risk of bias analysis are shown in Fig 3. This review clearly confirmed that methodological details of animal experiments are often poorly reported. Items concerning randomisation of animal housing, blinding and randomisation of outcome assessment(s), and blinding of caregivers and investigators were rarely described, which attributed to the high percentage of unclear risk of bias for all of these items. Sixty-three percent of included studies reported on randomisation of animals across experimental groups. However, only three [7, 9, 36] of these studies provided information regarding the methods used to generate the allocation sequence. Twelve papers (63%) did not report any measures for blinding. In case there was any mentioning of blinding (7 studies, 37%), this usually concerned blinding of an outcome assessor handling the histologic evaluation. Only one study [40] explicitly mentioned blinding for all outcomes. None of the studies reported power/sample size calculations to justify their experimental group sizes. Low risk of bias was scored for similarity of the animals across experimental groups (18 studies, 95%), addressing incomplete outcome data (17 studies, 89%), selective outcome reporting (17 studies, 89%) and other contributors to bias (16 studies, 84%). High risk of bias was scored for three papers for the question “Was the study apparently free of other problems that could result in high risk of bias?”. This was due to the fact that one study [16] seemed to have recycled control data from one of their historical cohorts [10] without mentioning this in their methods section. The other two studies [34, 39] lacked sufficient description of their used measures and/or measures of error, which could lead to wrongful interpretation of the results.

### Indicators of mechanical anastomotic strength

#### Bursting pressure

**Fig 4:**
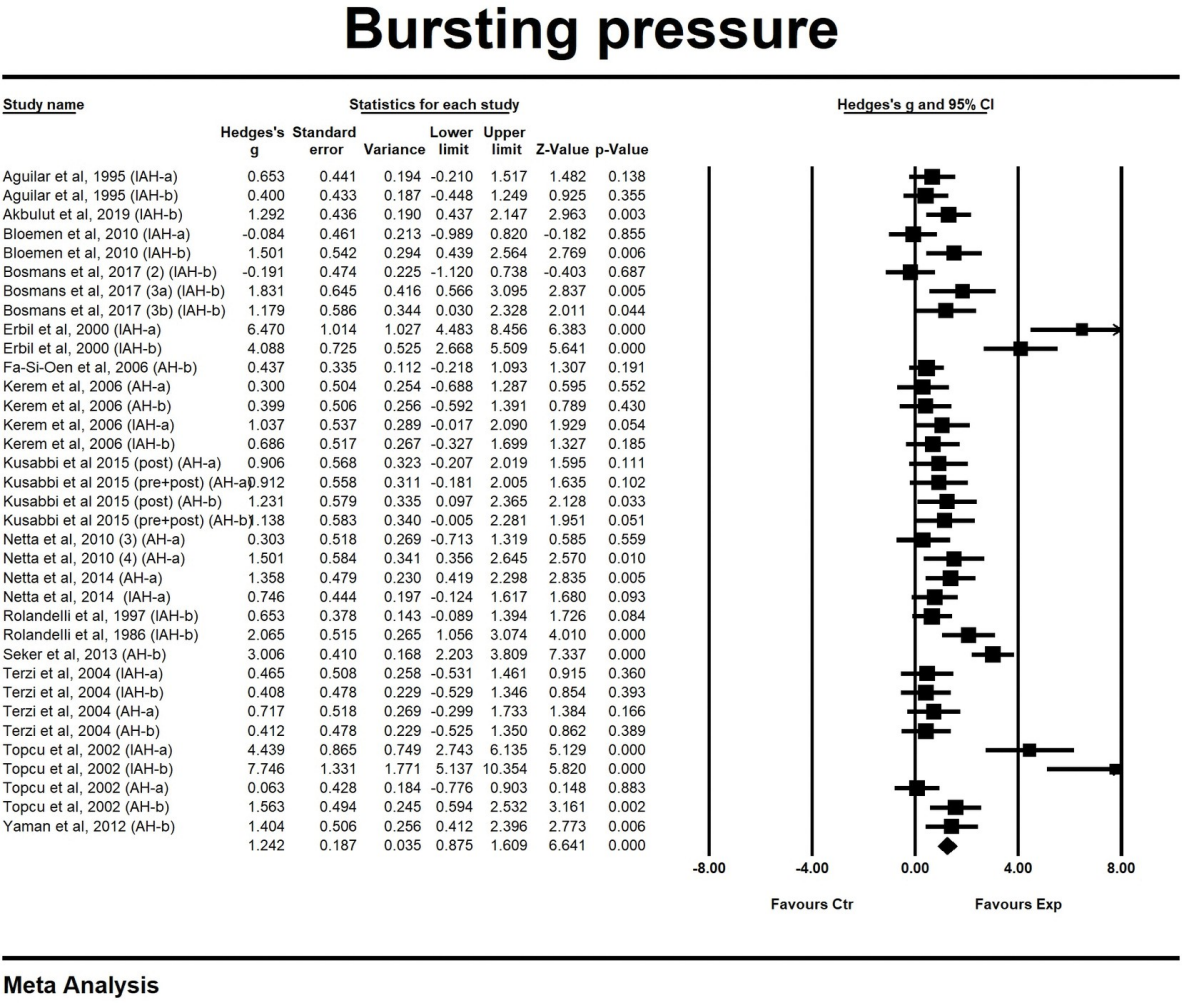
Forest plot of the effect of butyrate-containing applications on anastomotic bursting pressure.

Seventeen studies, containing 36 independent comparisons, investigated the effect of butyrate administration on the anastomotic bursting pressure in animal models for intestinal anastomosis. Of the seventeen studies, sixteen studies (35 comparisons) were pooled for meta-analysis. In these sixteen studies, overall administration of butyrate significantly increased bursting pressure by a SMD of 1.24 (95 per cent c.i. 0.88, 1.61), indicating an increased mechanical anastomotic strength (Fig 4). The study of Mathew and colleagues (2010) [34], which could not be included in the meta-analysis, tested the effect of postoperative administration of butyrate enemas in rats that were deprived of endogenous butyrate due to a fibre-free diet and a cecostomy (IAH). This study, containing only one comparison, showed no difference in BPR for anastomoses that were treated with 4 days of butyrate containing enemas after surgery compared to anastomoses that were treated with only saline containing enemas. Overall heterogeneity between pooled studies was high (I^2^ = 78%). Subgroup analyses were only conducted in groups with 5 studies or at least 10 independent comparisons. Consequently, subgroup analyses could only be performed for anastomotic model (AH or IAH) and timing of the assessment (a or b). Both subgroup analyses did not show any significant difference between subgroups (Tables 3 and 4). Butyrate increased anastomotic bursting pressure in both animal models of anastomotic healing (SMD 0.98, 0.44 to 1.52, n = 16) and models of impaired anastomotic healing (SMD 1.49, 0.98 to 2.00, n = 19). Studies that assessed the effect of butyrate administration on bursting pressure within 5 days postoperative (a) (SMD 1.10, 0.54 to 1.67, n = 15) or on day 5 and later (b) (SMD 1.35, 0.86 to 1.83, n = 20) both showed an increase in mechanical anastomotic strength.

**Table 3:** Effect of butyrate administration on bursting pressure in both animal models for intestinal anastomosis.

Anastomotic model	N	ES	LL	UL	*p-*value	I^2^
**AH**	16	0.98	0.44	1.52	0.000	64.3
**IAH**	19	1.49	0.98	2.00	0.000	84.3

**Table 4:** Early and late effects of butyrate administration on bursting pressure in animal models for intestinal anastomosis.

Timing of outcome assessment	N	ES	LL	UL	*p-*value	I^2^
**a**	15	1.10	0.54	1.67	0.000	77.1
**b**	20	1.35	0.86	1.83	0.000	79.4

Four studies used additional metrics to study the effect of butyrate administration on mechanical anastomotic strength. These studies calculated the force responsible for the loss of anastomotic continuity from LaPlace’s equation using the BPR and AC. This calculation resulted in the BWT, which significantly increased in three of four studies (Table 2). In the three studies that mentioned the size of the AC, AC size did not differ between butyrate treated and control treated anastomoses (Table 2).

#### Bursting site

**Fig 5:**
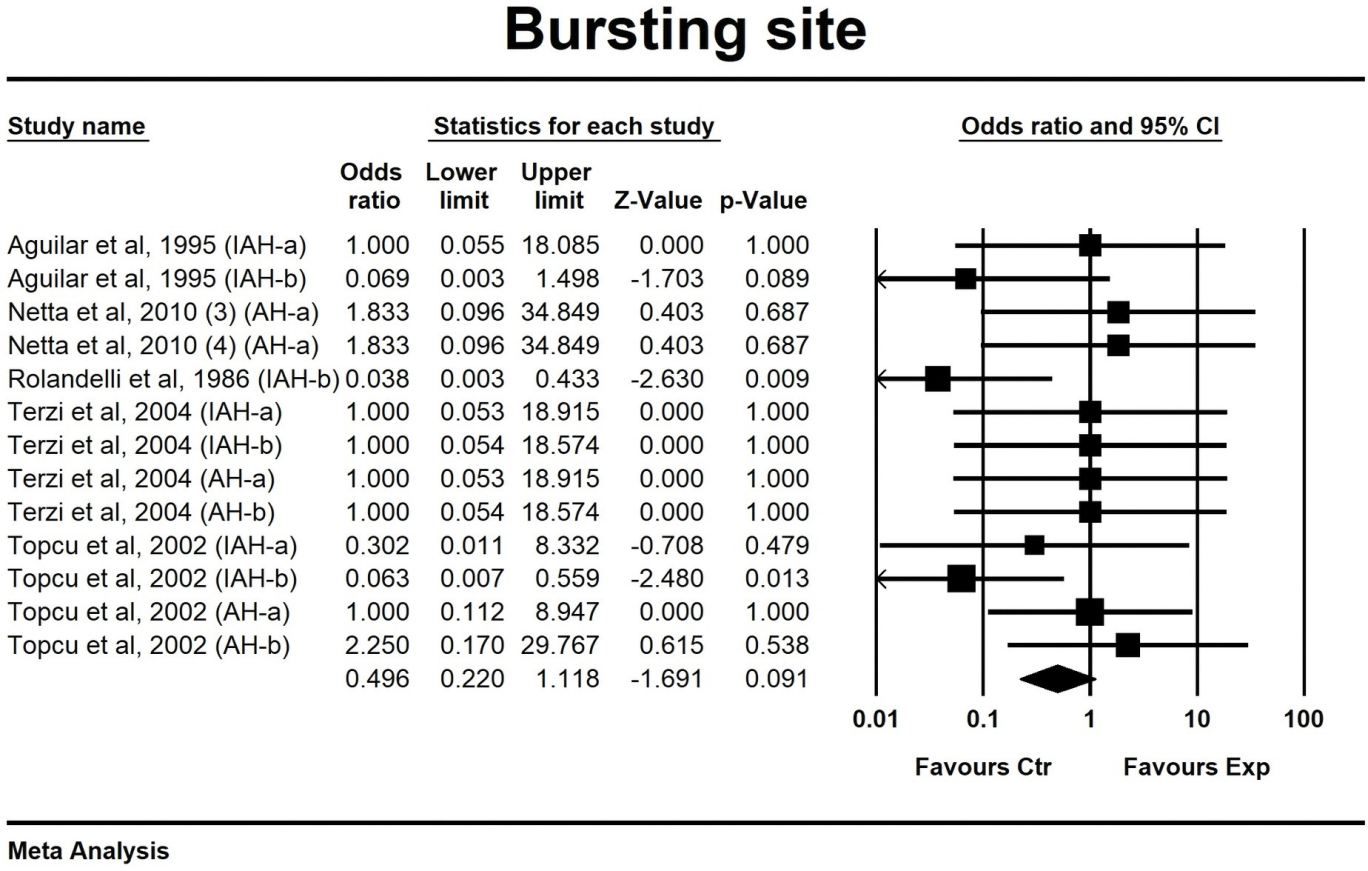
Forest plot of the effect of butyrate-containing applications on the probability of bursting in the suture line.

Five studies, containing 13 independent comparisons, investigated the effect of butyrate administration on the location of bursting (in the suture line vs outside the suture line) in animal models for intestinal anastomosis. Overall, for administration of butyrate (OR 0.50, 0.22 to 1.12) no clear effect on anastomotic strength could be observed (Fig 5). The overall heterogeneity between the studies was low (I^2^ = 12%). Subgroup analysis for anastomotic model and analysis of early (a) and late (b) effects of butyrate administration could not be conducted due to the low number of studies in each of the subgroups.

### Macroscopic indicators of anastomotic failure or other complications

#### Anastomotic leakage

**Fig 6:**
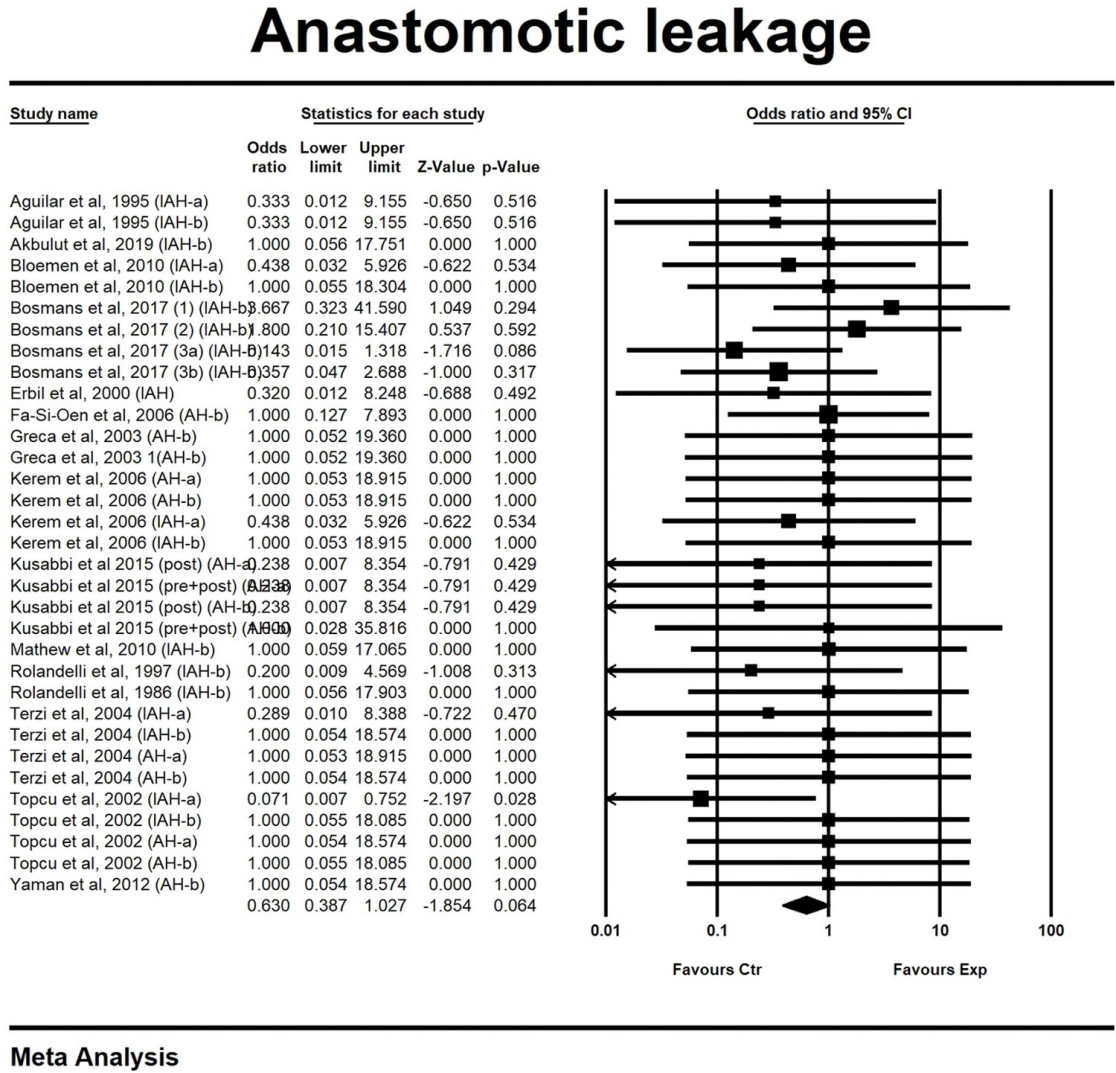
Forest plot of the effect of butyrate-containing applications on the probability of developing a spontaneous anastomotic leak.

A total of sixteen studies, containing 35 comparisons, investigated the effect of butyrate administration on the incidence of AL in animal models for intestinal anastomosis. Of sixteen studies, fifteen studies (33 comparisons) were pooled for meta-analysis (Fig 6). In these fifteen studies, overall administration of butyrate did not decrease odds of AL (OR 0.63, 0.39 to 1.03). The study that could not be included in the meta-analysis [10], containing two comparisons, also showed a decreased incidence of AL for butyrate treated anastomoses, but only when administered through colonic lavage. In the same study no clear effect on AL incidence could be observed in rats that were fed a butyrate-rich diet. Overall heterogeneity between pooled studies was low (I^2^ = 0%). Subgroup analyses were performed for both influence of anastomotic model and outcome effect timing on the overall effect of butyrate on AL incidence. First analysis (Table 5) showed that the effect of butyrate on AL incidence was not significantly different in AH (SMD 0.81, 0.37 to 1.78, n = 14) and IAH (SMD 0.54, 0.29 to 1.01, n = 19) models, however anastomoses in IAH models seemed to benefit slightly more from butyrate interventions. Interestingly, the second subgroup analysis (Table 6) separating the first 4 days (a) of healing from day 5 (b) and above, revealed that the effect of butyrate on AL was larger in group a (SMD 0.37, 0.17 to 0.93, n = 10) than in group b (SMD 0.80, 0.44 to 1.42, n = 22).

**Table 5:** Effect of butyrate administration on AL incidence in both animal models for intestinal anastomosis.

Anastomotic model	N	ES	LL	UL	*p-*value	I^2^
**AH**	14	0.81	0.37	1.78	0.598	00.0
**IAH**	19	0.54	0.29	1.01	0.052	00.0

**Table 6:** Early and late effects of butyrate administration on AL incidence in animal models for intestinal anastomosis.

Timing of outcome assessment	N	ES	LL	UL	*p-*value	I^2^
**a**	10	0.37	0.15	0.93	0.035	00.0
**b**	22	0.80	0.44	1.42	0.044	00.0

#### Adhesions

Nine studies, containing 27 comparisons, investigated the effect of butyrate administration on the incidence of intra-abdominal adhesions in animal models for intestinal anastomosis (Table 2). No meta-analysis could be conducted for this outcome measure. In seven of these studies (24 comparisons), no difference in adhesion formation was detected when comparing butyrate and control groups. Two studies (3 comparisons), however, did observe a significant decrease in the incidence of adhesions due to administration of butyrate. Netta and colleagues (2010) [10], who examined the effect of both a diet rich in butyrate and intraoperative SCFA lavage, found that SCFA lavage during surgery decreased the formation of adhesions after surgery in animal models for AH. Topcu and colleagues (2002) [39] observed a similar effect on adhesion formation in rats with colonic ischemia (IAH). These rats had significantly less intra-abdominal adhesions when administered an intraoperative enema containing a mix of SCFAs.

### Biochemical and histological indicators of anastomotic healing

#### Hydroxyproline/collagen

**Fig 7:**
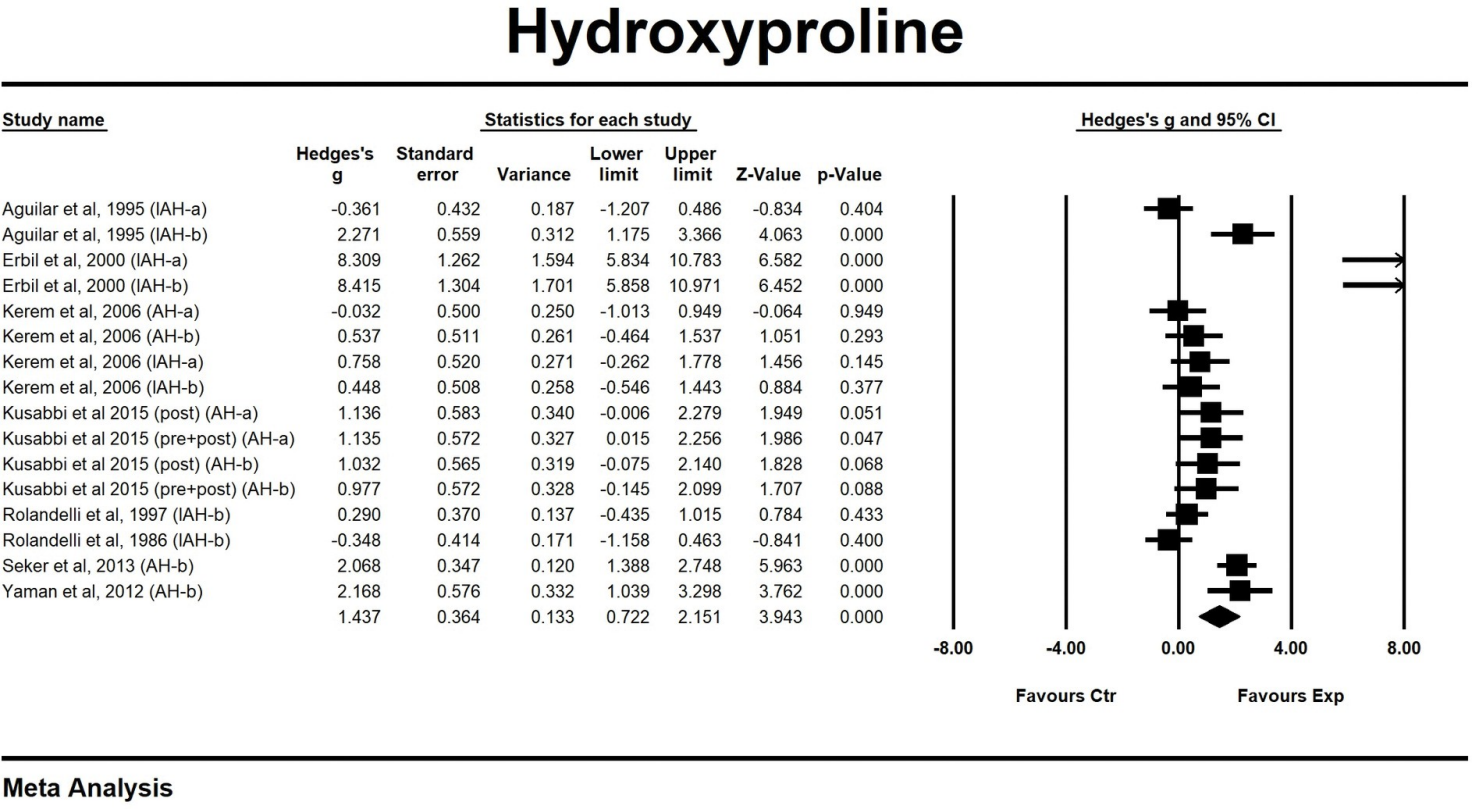
Forest plot of the effect of butyrate-containing applications on hydroxyproline content.

Eight studies, containing 16 comparisons, investigated the effect of butyrate administration on the anastomotic hydroxyproline content in animal models for intestinal anastomosis. Hydroxyproline content was assessed as an index of collagen synthesis and deposition. Overall the administration of butyrate significantly increased hydroxyproline content of the anastomoses by a SMD of 1.44 (95 per cent c.i. 0.72, 2.15), indicating an increase in collagen content due to butyrate (Fig 7). Heterogeneity between the studies was high (I^2^ = 87%). Subgroups were too small to conduct meaningful analyses.

Effect of butyrate on collagen metabolism and deposition was also evaluated using other methods. Two studies (Bloemen et al, 2010 & Bosmans et al, 2017) quantified the relative collagen areas in the anastomotic region as a percentage of total tissue surface and made a distinction between immature and mature collagen. Using this method, Bloemen and colleagues [7] found that a daily administration of butyrate enemas after surgery resulted in an increased mature-to-immature collagen ratio in the anastomotic tissue of rats in a defective suture model (IAH). The effect was seen as early as 3 days after the index surgery and by day 7 collagen ratios were similar between the butyrate and placebo enema groups. Bosmans and colleagues [9], who only tested this outcome at day 7, did not see a difference in the percentage of collagen deposition in all three experiments, nor a difference in the mature-to-immature collagen ratios. Greca and colleagues (2003) [26], however, did find an increase in collagen maturation on day 7 following a daily administration of butyrate enemas in rats (AH). This relative increase in mature collagen in the anastomosis was still present at 14 days after index surgery. Other studies that found an effect of butyrate administration on collagen deposition in anastomoses were the studies from Yaman (2012) [40] and Kusabbi (2015) [33]. In both studies an increase in collagen deposition in the anastomotic area was observed after a daily (before and/or after surgery) administration of AGHMB, a butyrate based oral nutritional supplement. These effects were seen as early as day 3 and lasted at least until 7 days after the index surgery.

MMPs, as important regulators of collagen metabolism, were analysed in three studies [7, 9, 32] (10 comparisons). No effect on MMP activity was found in the study of Bosmans and colleagues (2017), which evaluated the regenerative potential of butyrin-eluting patches (1), a single dose of intraluminal hyaluronan-butyrate during surgery (2) and postoperative daily administration of enemas containing either hyaluronan-butyrate (3a) or butyrate (3b) in rat models of IAH. The remaining two studies (Bloemen, et al 2010 & Kerem, et al 2006) found changes in MMP activity, but in opposite directions. SCFA enemas decreased MMP activity in rats receiving radiotherapy in the study of Kerem and colleagues, whereas butyrate enemas increased MMP activity in the study of Bloemen and colleagues.

#### Histological evaluation of wound healing

Eight studies, containing 22 comparisons, investigated the effect of butyrate administration on intestinal histology of the anastomotic tissue. No meta-analysis could be conducted for this outcome measure, therefore a summary of the findings is presented in Table 2. Most studies observed that butyrate administration did not affect the infiltration of immune cells into the anastomotic area, however, inflammatory cell infiltration was found to increase under the influence of butyrate in three studies. Terzi and colleagues (2004) [38] found that a 5 day preoperative administration of SCFA enemas resulted in an increased presence of granulocytes and macrophages 7 days after the index surgery (AH). Increased inflammatory cell infiltration was accompanied by an increase in reepithelialisation and granulation tissue. These effects were not observed in the anastomoses of the rats that were sacrificed and analysed at day 3. In the study of Kerem and colleagues (2006) [32], which tested the effect of SCFAs on anastomotic healing in both rats receiving preoperative radiotherapy (IAH) and rats not receiving preoperative radiotherapy (AH), the infiltration of granulocytes in the anastomotic area increased after a 5 day preoperative administration of SCFA enemas in rats receiving radiotherapy. This effect was only detected 7 days after surgery, which was accompanied by a decrease in necrosis, and an increase in reepithelialisation, fibroblast proliferation and granulation tissue. The histological evaluation of the rats that were sacrificed at day 3 postoperative only showed a similar effect for necrosis and granulation tissue. Terzi [38], in their additional experiment regarding anastomotic healing and radiotherapy (IAH), concluded the same on the effect of SCFAs on necrosis and granulation tissue in irradiated anastomoses. The third study [33] that found an increase in inflammatory cell infiltration, also showed an increase in neovascularization and fibroblast proliferation in almost all experimental groups. This study, by Kusabbi and colleagues (2015) [33], investigated the effect of an oral nutritional supplement containing AGHMB on anastomotic healing in rats. Other groups that investigated the effect of AGHMB on anastomotic healing found a similar effect on neovascularization and fibroblast proliferation 7 days postoperative (Akbulut et al, 2019) [29], and saw an increase in reepithelialisation (Yaman et al, 2012) [40]. Reepithelialisation of necrotic ulcers at the suture line, and deeper crypts, were also major findings in the study by Aguilar and colleagues (1997) [28] into the effects of intraoperative SCFA lavage on anastomotic healing in rats with colonic obstruction (IAH).

#### Cytokines

No meta-analysis could be conducted for this outcome measure. Three studies, containing 5 comparisons, investigated the effect of butyrate administration on cytokine production in animal models for intestinal anastomosis. Netta and colleagues (2010 & 2014) [10, 16] found that at 3 days postoperative TNF-α levels were lower due to intraoperative SCFA lavage in both rats receiving corticosteroids (IAH) and rats not receiving corticosteroids (AH) when compared to their controls. In the study of Akbulut and colleagues (2019) [29], no significant effect on TGF-β1 expression could be detected in immunosuppressed rats that received oral supplementation with AGHMB daily before and after surgery.

### Publication bias

Inspection of funnel plots for bursting pressure and anastomotic leakage did not show asymmetry. Duval and Tweedie’s Trim and Fill analysis confirmed this and showed no extra data points, which indicates that there was no significant overestimation of the observed effects due to publication bias.

## Discussion

This systematic review and meta-analysis evaluated the effect of butyrate on anastomotic healing in animal models of intestinal anastomosis. The main goal was to obtain insight into the potential clinical usefulness of butyrate-based interventions to prevent AL and to form a scientific basis for the development of new interventions or research into the subject. The analyses showed that administration of butyrate-containing applications significantly increases anastomotic strength which could have been caused by the observed increase of collagen synthesis and collagen maturation. The effects of butyrate on anastomotic healing were already detected within the first 4 days of anastomotic healing resulting in a significant decrease of AL incidence in this specific period. Additionally, the butyrate-induced increase in anastomotic strength coincided with a slightly decreased probability of bursting along the suture line. Subgroup analysis for type of anastomotic model, AH or IAH, did not reveal significant differences in the effect of butyrate on anastomotic healing. Unfortunately, the number of studies for each specific type of butyrate was too restricted to conduct a meaningful analysis, and therefore no conclusion could be drawn about the effectivity of each of the individual compounds. Nevertheless, there are indications that almost all forms of butyrate exert a positive effect and that even a single intraluminal application of a SCFA mixture during surgery can already have a large effect on HP content and BPR [30, 39].

Anastomotic BPR is a frequently used marker of anastomotic healing in animal models and one of the primary outcomes of this review [22]. Although its use has been debated for disrupting tissue samples and being more dependent on the suturing technique than on the actual intervention itself [4, 22], it is considered as an appropriate marker for anastomotic strength [21]. In the first days of anastomotic healing, collagen degradation typically exceeds collagen synthesis, which makes newly constructed anastomoses vulnerable and less resistant to mechanical stress [16]. Arguably, this lack of anastomotic strength makes anastomoses especially prone to leakage in these first days, hence the importance of strength testing when evaluating the efficacy of new therapies. The aforementioned also explains the choice for subgroup analyses of all study comparisons containing data collected within the first 4 days after surgery, and comparisons containing data of day 5 or later. In theory, interventions should ideally already demonstrate some effect in the early phase of healing in order to prevent actual problems later on. From the 19 articles that were evaluated for this review, 17 (35 comparisons) provided data regarding the strength of the anastomosis of which 9 studies (15 comparisons) had data that was collected in the first 4 postoperative days and 14 studies (20 comparisons) with data from day 5 or later. Butyrate administration resulted in a significant increase in BPR in each of these analyses and in both anastomotic models, which makes the positive effect of butyrate on anastomotic strength solid.

Another indicator of anastomotic strength that was used for this review was bursting site. The location of the rupture during BPR testing shows us where the weaknesses are in that particular intestinal segment. As the average healed wound reaches only approximately 80% of the strength of uninjured tissue near the end of the remodelling phase [41], anastomoses typically remain the Achilles’ heel of the intestine and are likely the first to rupture during pressure testing. The bursting site data of this review, unfortunately, showed no difference in incidence of anastomotic bursts between both groups meaning that butyrate was not able to decrease the aforementioned relative tissue weakness. However, timing of the assessment might have an important influence on the overall effect as collagen maturation and remodelling usually does not start before day 5 after surgery in rats.

In theory, signs of anastomotic failure would be the most valuable as primary outcome, because this outcome best reflects clinical situation [4]. Yet, not all studies on anastomotic healing report AL rates or use an appropriate definition of AL [42]. Sixteen out of 19 articles reported AL (84 per cent) and only half of those (8 studies) used AL rates to compare groups. From the 8 studies that incorporated AL in their outcome testing, only 3 studies scored the severity of the leaks. Analysing this data, no significant difference in spontaneous dehiscence between butyrate and control groups could be determined. A possible explanation for this finding could be the fact that rats have a high tendency to heal without significant problems, including in gastrointestinal anastomoses [7, 43]. This hypothesis is somewhat supported by the difference in AL rates between AH and IAH groups, because rats with impaired healing benefitted slightly more from butyrate administration than rats in normal healing experiments. The results of the subgroup analysis on the effect of time, interestingly, showed that butyrate administration does lead to a decreased AL incidence in the first four days after surgery. Knowing that anastomoses are extra susceptible to dehiscence in these early days, this finding is of particular interest. Fortunately, administration of butyrate did not cause an increase in adhesions or anastomotic stricture. Butyrin-eluting patches, however, caused mechanical obstruction of the intestinal lumen which negatively impacted anastomotic healing [9].

Collagen fibres throughout the intestinal tissue are responsible for most of the strength of an anastomosis. The metabolism of collagen, although assessed in multiple ways, was overall positively influenced by butyrate administration, explaining the elevated bursting resistance and shift in bursting site. Positive effects seem to stem from alterations of collagen breakdown, as measured by MMP activity, and stimulation of collagen synthesis, as measured by hydroxyproline content. Additionally, maturation of the newly synthesized collagen fibres was found to be accelerated upon butyrate administration, reducing the window of vulnerability for newly constructed anastomoses.

Histological parameters of wound healing are often reported in experimental studies regarding intestinal anastomoses. These parameters, like inflammatory cell infiltration, fibroblast proliferation, collagen deposition and neovascularization, are usually based on cutaneous wound healing, but are helpful in evaluating the general wound healing process at the anastomotic site [22]. In the narrative analysis on histological effects of butyrate administration on anastomotic healing, no apparent effect of butyrate on general wound healing parameters was found. Nevertheless, some studies [28, 29, 32, 33, 38, 40] demonstrated positive effects on inflammatory cell infiltration, reepithelialisation, fibroblast proliferation and neovascularisation, of which some have also been mentioned in the latest review of Hajjar and colleagues (2021) [18] concerning the role of butyrate in colorectal cancer surgery. The lack of a clear and consistent conclusion in this review might have been caused by the limited amount of studies included and the substantial heterogeneity between the studies.

Finally, this review is limited by a relatively high number of poor- to fair-quality studies, as revealed in the risk of bias analysis. Essential details concerning the used AL definitions, experimental designs and conduct were often poorly reported, resulting in an unclear risk of bias. Although marginal reporting is common in experimental animal research, this does affect the ability to draw reliable conclusions from the included studies. Another limitation is the considerable degree of heterogeneity among the studies induced by a large variability between the types of butyrate-based interventions used, animal models, administration route, dose and timing of outcome assessment. Although some of the heterogeneity is necessary to assess treatment efficacy under specific conditions, greater similarity between studies would allow better assessment of external validity [4, 21]. A different limitation concerns the use of surrogate markers to assess anastomotic healing. Anastomotic healing is a complex and dynamic process involving many different biological processes that are difficult to assess altogether. For this reason, the results of animal studies into intestinal anastomosis were compared using surrogate outcomes of anastomotic healing that resemble clinical phenomena or focus on general parameters of wound healing. Results should therefore be interpreted with caution, as they might not directly translate into true anastomotic healing or clinical situation. Lastly, the clinical relevance of the included models is somewhat debatable. As mentioned before, rats are less likely to develop anastomotic leaks as they naturally tend to heal without major complications [7, 43]. As a consequence, rat models might not be the most appropriate to test hypotheses concerning anastomotic leakage/healing. Most of the studies used for this review, however, included IAH models to increase the comparability between rats and humans. Another concern regarding the relevance of the included models is that all studies used animals that were cancer free. In the clinical situation, most patients undergoing restorative intestinal resections have cancer, which may confound the results related to the used animal models. In addition to this, almost all animals were male. Even though clinical research has shown a higher AL risk for men [44], both women and men are susceptible to develop AL. Results of included studies may therefore not be as applicable to women as they are to men. A more practical issue of the used models is administration route. Most studies used delivery methods that would not be appropriate for clinical use. Enemas, for example, are considered too invasive for patients recovering from digestive tract surgery, as their administration could potentially damage newly formed anastomoses. Also, the unpleasant taste and odour of butyrate hinders oral administration [9]. Indirect supplementation of SCFAs through fiber-rich foods could be a feasible alternative, however, these depend on microbial metabolism in order to be transformed into SCFAs [32]. Exogenous supplementation, on the contrary, is better controlled and less dependent on the intestinal microenvironment for its effect. It is due to these arguments that indirect supplementation has not been a topic of this systematic review.

Altogether, butyrate seems to be effective in increasing anastomotic resilience in animal models for anastomotic leakage/healing, which suggests that there is potential ground to investigate the use of butyrate-based interventions in future clinical trials for AL. Before clinical translation, however, it is appropriate to first conduct some additional pre-clinical research to compare each of the compounds in identical models and determine the best administration route, dose and frequency. Once these trials have been completed, we feel that in order to minimise unnecessary further animal testing on this subject, the evidence should be used to fuel translational research.

## Supporting information

S1 Table Containing the search strategy. (DOCX)

S1 Checklist PRISMA checklist. (DOC)

## References

[1] Garude K, Tandel C, Rao S, Shah N. Single Layered Intestinal Anastomosis: A Safe and Economic Technique. Indian Journal of Surgery. 2013;75. doi: 10.1007/s12262-012-0487-7 24426455 PMC3726811

[2] El-Badawy H. Anastomotic Leakage After Gastrointestinal Surgery: Risk Factors, Presentation And Outcome. 2014;57(October):494–512. doi: 10.12816/0008484

[3] Praagh JBV, Goffau MCD, Bakker IS, Harmsen HJM, Olinga P, Havenga K. Intestinal microbiota and anastomotic leakage of stapled colorectal anastomoses: a pilot study. Surgical Endoscopy. 2016;30(6):2259–65. doi: 10.1007/s00464-015-4508-z 26385781 PMC4887536

[4] Yauw ST, Wever KE, Hoesseini A, Ritskes-Hoitinga M, van Goor H. Systematic review of experimental studies on intestinal anastomosis. Br J Surg. 2015;102(7):726–34. doi: 10.1002/bjs.9776 25846745

[5] Law WL, Choi HK, Lee YM, Ho JW, Seto CL. Anastomotic leakage is associated with poor long-term outcome in patients after curative colorectal resection for malignancy. J Gastrointest Surg. 2007;11(1):8–15. doi: 10.1007/s11605-006-0049-z 17390180

[6] McDermott FD, Heeney A, Kelly ME, Steele RJ, Carlson GL, Winter DC. Systematic review of preoperative, intraoperative and postoperative risk factors for colorectal anastomotic leaks. British Journal of Surgery. 2015;102(5):462–79. doi: 10.1002/bjs.9697 25703524

[7] Bloemen JG, Schreinemacher MH, De Bruine AP, Buurman WA, Bouvy ND, Dejong CH. Butyrate enemas improve intestinal anastomotic strength in a rat model. Diseases of the Colon and Rectum. 2010;53(7):1069–75. doi: 10.1007/DCR.0b013e3181d881b7 20551761

[8] Chiarello MM, Fransvea P, Cariati M, Adams NJ, Bianchi V, Brisinda G. Anastomotic leakage in colorectal cancer surgery. Surg Oncol. 2022;40:101708. doi: 10.1016/j.suronc.2022.101708 35092916

[9] Bosmans JW, Jongen AC, Boonen BT, van Rijn S, Scognamiglio F, Stucchi L, et al. Comparison of three different application routes of butyrate to improve colonic anastomotic strength in rats. International journal of colorectal disease. 2017;32:305–13. doi: 10.1007/s00384-016-2718-z 27942836 PMC5316396

[10] Netta S, Michalopoulos A, Apostolidis S, Paramythiotis D, Papavramidis T, Papadopoulos V, et al. Enhancement of colonic anastomotic strength in rats by short-chain fatty acids. Techniques in Coloproctology. 2010;14:53–5. doi: 10.1007/s10151-010-0611-2 20683753

[11] Dou X, Gao N, Yan D. Sodium Butyrate Alleviates Mouse Colitis by Regulating Gut Microbiota Dysbiosis. 2020. 10.3390/ani10071154 PMC7401615 32645998

[12] Pacheco RG, Esposito CC, Müller LCM, Castelo-branco MTL, Quintella LP, Chagas VLA, et al. Use of butyrate or glutamine in enema solution reduces inflammation and fibrosis in experimental diversion colitis. 2012;18(32):4278–87. doi: 10.3748/wjg.v18.i32.4278 22969190 PMC3436042

[13] Bach Knudsen Ke SACNJKS. New insight into butyrate metabolism. Proc Nutr Soc. 2003;62(1):81–6. doi: 10.1079/PNS2002212 12740062

[14] Wu X, Wu Y, He L, Wu L, Wang X, Liu Z. Effects of the intestinal microbial metabolite butyrate on the development of colorectal cancer. Journal of Cancer. 2018;9. doi: 10.7150/jca.25324 30026849 PMC6036887

[15] Gonçalves P, Martel F. Butyrate and colorectal cancer: the role of butyrate transport. Curr Drug Metab. 2013;14(9):994–1008. doi: 10.2174/1389200211314090006 24160296

[16] Netta S, Papadopoulos VN, Apostolidis S, Michalopoulos A. The effect of intraoperative lavage with short chain fatty acids (SCFAs) on rectal anastomosis of rats receiving corticosteroids. Hippokratia. 2014;18(4):350–4. 26052203 PMC4453810

[17] Braskén P. Healing of experimental colon anastomosis. Eur J Surg Suppl. 1991(566):1–51. 1725603

[18] Hajjar R, Richard CS, Santos MM. The role of butyrate in surgical and oncological outcomes in colorectal cancer. Am J Physiol Gastrointest Liver Physiol. 2021;320(4):G601–g8. doi: 10.1152/ajpgi.00316.2020 33404375 PMC8238168

[19] Liberati A, Altman DG, Tetzlaff J, Mulrow C, Gøtzsche PC, Ioannidis J. The PRISMA statement for reporting systematic reviews and meta-analyses of studies that evaluate health care interventions: explanation and elaboration. Journal of clinical epidemiology. 2009;62(10):e1–e34. doi: 10.1016/j.jclinepi.2009.06.006 19631507

[20] Ouzzani M, Hammady H, Fedorowicz Z, Elmagarmid A. Rayyan-a web and mobile app for systematic reviews. Syst Rev. 2016;5(1):210. doi: 10.1186/s13643-016-0384-4 27919275 PMC5139140

[21] Bosmans J, Moossdorff M, Al-Taher M, van Beek L, Derikx JPM, Bouvy ND. International consensus statement regarding the use of animal models for research on anastomoses in the lower gastrointestinal tract. Int J Colorectal Dis. 2016;31(5):1021–30. doi: 10.1007/s00384-016-2550-5 26960997 PMC4834109

[22] Bosmans JW, Jongen AC, Bouvy ND, Derikx JP. Colorectal anastomotic healing: why the biological processes that lead to anastomotic leakage should be revealed prior to conducting intervention studies. BMC Gastroenterol. 2015;15:180. doi: 10.1186/s12876-015-0410-3 26691961 PMC4687306

[23] Rohatgi A. WebPlotDigitizer Pacifica, CA, USA2021 [Available from: https://automeris.io/WebPlotDigitizer/.

[24] Hooijmans CR, Rovers MM, de Vries RB, Leenaars M, Ritskes-Hoitinga M, Langendam MW. SYRCLE’s risk of bias tool for animal studies. BMC Med Res Methodol. 2014;14:43. doi: 10.1186/1471-2288-14-43 24667063 PMC4230647

[25] Hooijmans CR, Draper D, Ergun M, Scheffer GJ. The effect of analgesics on stimulus evoked pain-like behaviour in animal models for chemotherapy induced peripheral neuropathy- a meta-analysis. Sci Rep. 2019;9(1):17549. doi: 10.1038/s41598-019-54152-8 31772391 PMC6879539

[26] Hintz Greca F, de Lourdes Pessole Biondo-Simões M, Dello Monaco Martins V, Henrique de Araújo F, Buzetti Milano J. A ácidos graxos de cadeia curta na cicatrização de anastomoses colônicas: estudo experimental em ratos. Revista do Colégio Brasileiro de Cirurgiões. 2003;30(4):268–74.

[27] Aguilar-Nascimento JE, Mathie RT, Man WK, Williamson RC. Enhanced intra-anastomotic healing by operative lavage with nutrient solutions in experimental left-sided colonic obstruction. The British journal of surgery. 1995;82(4):461–4. doi: 10.1002/bjs.1800820410 7613886

[28] Aguilar-Nascimento JE, Oliveira-Neto JP, Mathie RT, Williamson RC. Effect of intraoperative nutritional solutions on perianastomotic colonic mucosa in experimental large bowel obstruction. Digestive diseases and sciences. 1997;42(12):2581–4. doi: 10.1023/a:1018837301224 9440641

[29] Akbulut S, Dogan Z, Baskiran A, Elbe H, Turkoz Y. Effect of a honey and arginine-glutamine-hydroxymethylbutyrate mixture on the healing of colon anastomosis in rats immunosuppressed with tacrolimus. Biotech Histochem. 2019;94(7):514–21. doi: 10.1080/10520295.2019.1601257 30983411

[30] Erbil Y, Calis A, Berber E, Mercan S. The effect of intraoperative colonic lavage with NG-nitro-L-arginine methyl ester (L-NAME) on anastomotic healing in the presence of left-sided colonic obstruction in the rat. Surgery today. 2000;30:421–5. doi: 10.1007/s005950050615 10819477

[31] Fa-Si-Oen P, Van De Gender P, Putter H, Ectors N, D’Hoore A, Topal B, et al. The effect of polyethylene glycol and butyrate on anastomotic healing in the rat colon. Techniques in Coloproctology. 2006;10(4):308–11. doi: 10.1007/s10151-006-0298-6 17115318

[32] Kerem M, Bedirli A, Karahacioglu E, Pasaoglu H, Sahin O, Bayraktar N, et al. Effects of soluble fiber on matrix metalloproteinase-2 activity and healing of colon anastomosis in rats given radiotherapy. Clin Nutr. 2006;25(4):661–70. doi: 10.1016/j.clnu.2006.01.028 16677740

[33] Kusabbi R, Kismet K, Kuru S, Barlas AM, Duymus ME, Hasanoglu A, et al. Effects of the Oral Nutritional Supplement Containing Arginine, Glutamine, and Hydroxymethylbutyrate (Abound®) on Healing of Colonic Anastomoses in Rats. Indian J Surg. 2015;77:1242–7. 27011545 10.1007/s12262-015-1268-x PMC4775590

[34] Mathew AJ, Wann VC, Abraham DT, Jacob PM, Selvan BS, Ramakrishna BS, et al. The effect of butyrate on the healing of colonic anastomoses in rats. Journal of Investigative Surgery. 2010;23(2):101–4. doi: 10.3109/08941930903469367 20497012

[35] Rolandelli RH, Buckmire MA, Bernstein KA. Intravenous butyrate and healing of colonic anastomoses in the rat. Diseases of the Colon and Rectum. 1997;40(1):67–70. doi: 10.1007/BF02055684 9102264

[36] Rolandelli RH, Koruda MJ, Settle RG, Rombeau JL. Effects of intraluminal infusion of short-chain fatty acids on the healing of colonic anastomosis in the rat. Surgery. 1986;100(2):198–204. 3738751

[37] Seker D, Ergil J, Ozkan D, Akinci M, Yalcindag A, Ginis Z, et al. The effects of supplementation with a mixture of arginine, glutamine, and beta-hydroxy beta-methylbutyrate on the healing of colon anastomoses. Acta Chir Belg. 2013;113(6):444–8. 24494473

[38] Terzi C, Sevinç AI, Koçdor H, Oktay G, Alanyali H, Küpelioğlu A, et al. Improvement of colonic healing by preoperative rectal irrigation with short-chain fatty acids in rats given radiotherapy. Diseases of the colon and rectum. 2004;47(12):2184–94. doi: 10.1007/s10350-004-0724-7 15657672

[39] Topcu O, Karadayi K, Kuzu MA, Ulukent S, Erkek B, Alacayir I. Enteral and intraluminal short-chain fatty acids improves ischemic left colonic anastomotic healing in the rat. International Journal of Colorectal Disease. 2002;17(3):171–6. doi: 10.1007/s003840100357 12049311

[40] Yaman I, Kara C, Derici H, Diniz G, Ortac R, Ozyurt BC. THE EFFECT OF SPECIALIZED AMINO ACID MIXTURE ON HEALING OF LEFT COLONIC ANASTOMOSIS: AN EXPERIMENTAL STUDY. Turkiye Klinikleri Journal of Medical Sciences. 2012;33(3):678–84. doi: 10.5336/medsci.2012-30385

[41] Lam A, Fleischer B, Alverdy J. The Biology of Anastomotic Healing-the Unknown Overwhelms the Known. J Gastrointest Surg. 2020;24(9):2160–6. doi: 10.1007/s11605-020-04680-w 32524361 PMC7446770

[42] van Helsdingen CP, Jongen AC, de Jonge WJ, Bouvy ND, Derikx JP. Consensus on the definition of colorectal anastomotic leakage: A modified Delphi study. World J Gastroenterol. 2020;26(23):3293–303. doi: 10.3748/wjg.v26.i23.3293 32684743 PMC7336323

[43] Komen N, van der Wal HC, Ditzel M, Kleinrensink GJ, Jeekel H, Lange JF. Colorectal anastomotic leakage: a new experimental model. J Surg Res. 2009;155(1):7–12. doi: 10.1016/j.jss.2008.08.019 19446852

[44] Huisman DE, Reudink M, van Rooijen SJ, Bootsma BT, van de Brug T, Stens J, et al. LekCheck: A Prospective Study to Identify Perioperative Modifiable Risk Factors for Anastomotic Leakage in Colorectal Surgery. Ann Surg. 2020;275(1):e189–e97. doi: 10.1097/SLA.0000000000003853 32511133 PMC8683256

