# Supplementary material for: Enhancing intestinal anastomotic healing using butyrate: Systematic review and meta-analysis of experimental animal studies
Source: PLoS One. 2023 Jun 13;18(6):e0286716. doi: 10.1371/journal.pone.0286716 (PMC10263344; doi:10.1371/journal.pone.0286716)
Supplement: S1 Table — (DOCX) [file pone.0286716.s001.docx]

**Table S1** Search strategy

| **PubMed search command (anastomosis + butyrate)** |
| --- |
| ((anastomo*[tiab] OR surgical anastomosis[MeSH]) OR "anastomotic leak"[MeSH])  **AND**  fatty acids, volatile[MeSH] OR butyrates[MeSH] OR butyric acid[MeSH] OR butyrat* [tiab] OR butyric acid* [tiab] OR “short-chain fatty acid*” [tiab] OR SCFA [tiab] OR butanoic [tiab] OR hydroxymethylbutyrate [tiab] OR HMB [tiab] OR “volatile fatty acid*” [tiab] |
| **Ovid Embase search command (anastomosis + butyrate)** |
| anastomo*.ti,ab,kw. or exp Anastomosis leakage/  **AND**  exp short chain fatty acid/ OR (butyrat* OR butyric acid* OR short-chain fatty acid* OR SCFA OR butanoic OR hydroxymethylbutyrate OR HMB OR volatile fatty acid*).ti,ab,kw. |
